# Undifferentiated-type predominant mixed-type early gastric cancer is a significant risk factor for requiring additional surgeries after endoscopic submucosal dissection

**DOI:** 10.1038/s41598-020-63781-3

**Published:** 2020-04-21

**Authors:** Yusuke Horiuchi, Junko Fujisaki, Noriko Yamamoto, Naoki Ishizuka, Akiyoshi Ishiyama, Toshiyuki Yoshio, Toshiaki Hirasawa, Yorimasa Yamamoto, Masatsugu Nagahama, Hiroshi Takahashi, Tomohiro Tsuchida

**Affiliations:** 1 Department of Gastroenterology, Cancer Institute Hospital, Tokyo, Japan; 2 Department of Pathology, Cancer Institute Hospital, Tokyo, Japan; 3 Department of Clinical Trial Planning and Management, Cancer Institute Hospital, Tokyo, Japan; 4 Department of Gastroenterology, Showa University Fujigaoka Hospital, Kanagawa, Japan

**Keywords:** Gastric cancer, Gastrointestinal cancer, Stomach diseases

## Abstract

We aimed to clarify the differences in therapeutic outcomes of patients with pure undifferentiated-type and mixed undifferentiated-type cancers who underwent endoscopic submucosal dissection (ESD), and whether pre-treatment diagnosis of mixed undifferentiated-type cancer is associated with requiring additional surgery after ESD. Patients subjected to ESD as initial treatment between May 2005 and March 2017 were enrolled. There were 277 undifferentiated-type cancers (265 patients). Histologically, 258 lesions were pure-type and 19 were mixed-type. We compared therapeutic outcomes and pre-treatment factors (tumour diameter, tumour depth, ulcerative findings, tumour location, and the macroscopic, and histological type of the biopsy specimen) between pure-type and mixed-type lesions, and between cases not requiring additional surgeries and cases requiring additional surgeries. Tumour diameter >20 mm, submucosal invasion, and the presence of ulcerative findings made pre-treatment diagnosis more difficult for mixed-type than for pure-type lesions. In cases requiring additional surgery, pre-treatment diagnosis of mixed-type lesions was significantly more likely than pre-treatment diagnosis of pure-type lesions. For mixed-type lesions, pre-treatment histological diagnosis and careful consideration are necessary to determine indications for ESD to avoid additional surgery after ESD.

## Introduction

Because of the development of endoscopic submucosal dissection (ESD) for early gastric cancer (EGC)^1–4^, gastric cancer lesions that previously required surgical treatment can now be resected using less invasive endoscopic procedures. However, the indications for ESD are limited. For differentiated-type (DT) EGC (according to the World Health Organization (WHO) classification^5^; tubular adenocarcinoma (well-differentiated, moderately differentiated), papillary adenocarcinoma, and according to the Japanese classification of gastric carcinoma^6^; tubular adenocarcinoma, well-differentiated [tub1], tubular adenocarcinoma, moderately differentiated [tub2], papillary adenocarcinoma [pap]), ESD is indicated only for intramucosal cancer with no ulcerative findings, or intramucosal cancer with a tumour diameter ≤30 mm and positive ulcerative findings^7,8^. For undifferentiated-type (UDT) EGC (according to the WHO classification^5^; poorly cohesive carcinoma, including signet ring cell carcinoma and other subtypes, and according to the Japanese classification of gastric carcinoma^6^, signet ring cell carcinoma [sig], poorly differentiated adenocarcinoma [non-solid type, por2]), ESD is indicated only for intramucosal cancer with a tumour diameter ≤20 mm and no ulcerative findings^7,9^. Lesions that do not meet these indications for ESD are treated with surgery.

In national guidelines using the WHO classification, there are no indication criteria or evaluation of curability for ESD in undifferentiated-type gastric cancer. The description exists only in Japanese guidelines that are used by Asian countries. Therefore, in this study we used Japanese guidelines, as our objective was to determine the therapeutic outcome of ESD for undifferentiated-type gastric cancer. However, to allow readers to contextualise the contents, we have provided information on the WHO classification.

Generally, the histological type of cancer is determined by performing a pre-treatment biopsy^7,10,11^. Mixed-type (MT) gastric cancer demonstrates histological components of both the differentiated type and the undifferentiated type. According to the Japanese guidelines, UDT-predominant MT (M-UDT) cancers should be considered UDT cancers^7^.

In surgical specimens of gastric cancer, it has been reported that the frequency of lymphovascular invasion and the risk of lymph node metastasis were higher in M-UDT gastric cancer than in pure UDT (P-UDT) gastric cancer, which demonstrates the greater malignancy potential of M-UDT gastric cancer^12–14^. However, reports on the difference between M-UDT and P-UDT in cases of ESD are not available.

Regarding DT cancers, we previously reported that DT-predominant MT (M-DT) EGC had a higher rate of additional surgeries after ESD than did pure DT (P-DT)^15^. This suggests that making a pre-treatment diagnosis to determine the indications for ESD in M-DT cancers is difficult. However, differences in therapeutic outcomes following ESD for P-UDT cancers and M-UDT cancers have not been reported, and the correlation of pre-treatment diagnosis of M-UDT with requiring additional surgeries after ESD has not been clarified.

According to the Japanese guidelines, in UDT cancers, endoscopic curability B (eCURA B) refers to curability not requiring additional surgeries after ESD, and endoscopic curability C2 (eCURA C2) refers to curability requiring additional surgeries after ESD^7^.

In this study, we aimed to clarify the differences in therapeutic outcomes of ESD for P-UDT cancers and M-UDT cancers and to clarify whether pre-treatment diagnosis of M-UDT is associated with eCURA C2.

## Results

**Figure 1:**
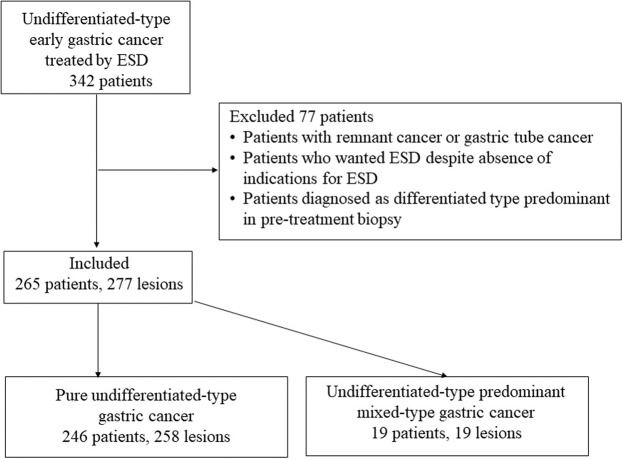
Flow diagram of patient enrolment, allocation, and analysis. ESD, endoscopic submucosal dissection.

Among patients who underwent ESD as initial treatment at our hospital between May 2005, and March 2017, 342 patients were diagnosed with UDT EGC. However, because some patients did not fulfil the inclusion criteria, 265 patients with 277 lesions were included in this study. Histologically, 246 patients with 258 lesions were P-UDT, and 19patients with 19 lesions were M-UDT (Fig. 1). Table 1 summarises the characteristics and therapeutic outcomes of all UDT EGC cases included in this study.

**Table 1:** Patient characteristics and therapeutic outcomes of endoscopic submucosal dissection for undifferentiated-type gastric cancer (265 patients, 277 lesions).

Characteristic	N (%)
Age, years	58 (48–67)30–86
Sex, male/female	154/111 (58.1/41.9)
Location	
Upper third	29 (10.5)
Middle third	181 (65.3)
Lower third	67 (24.2)
Macroscopic type	
Flat, depressed	271 (97.8)
Others(Complex, Elevated)	6 (2.2)
Pre-treatment histology based on biopsy findings	
P-UDT	270 (97.5)
M-UDT	7 (2.5)
**Therapeutic outcomes**	
Median tumour diameter, mm	10 (6–15) 1–40
≥20 mm	19 (6.9)
Invasion depth	
Intramucosal	252 (91.0)
Submucosa	25 (9.0)
Presence of lymphovascular invasion	
Lymphatic invasion	4 (1.4)
Vascular invasion	1 (0.3)
Positive vertical margin	4 (1.4)
Positive horizontal margin	7 (2.5)
Presence of ulcerative findings	14 (5.0)
En bloc resection	276 (99.6)
Post-treatment histological type	
P-UDT	258 (93.1)
M-UDT	19 (6.9)
eCURA B	229 (82.7)

Data are presented as number (%), except for age and tumour diameter, which are expressed as median (interquartile range) [range].

M-UDT, undifferentiated type predominant mixed type; P-UDT, pure undifferentiated type.

eCURA B was defined in accordance with the Japanese guidelines for intramucosal undifferentiated lesions measuring ≤20 mm in diameter, lesions without ulcerative findings, lesions with negative horizontal margins, lesions with negative vertical margins, and lesions without lymphovascular invasion^6^. eCURA B is refer to the curability not requiring additional surgeries after ESD.

We compared the post-treatment histological type associated with the cases of eCURA B and the cases of eCURA C2 who underwent ESD for UDT EGC (Table 2). Cases of eCURA B were significantly more likely to be achieved in P-UDT lesions than in M-UDT lesions.

**Table 2:** Comparison of post-treatment histological type between the cases of eCURA B and the cases of eCURA C2 treated with endoscopic submucosal dissection for undifferentiated-type early gastric cancer.

	eCURA B (n = 229)	eCURA C2 (n = 48)	P-value
Post-treatment histological type			0.0003
P-UDT	220 (85.3)	38 (14.7)	
M-UDT	9 (47.4)	10 (52.6)	

M-UDT, undifferentiated type predominant mixed type; P-UDT, pure undifferentiated type.

eCURA B was defined in accordance with the Japanese guidelines for intramucosal undifferentiated lesions measuring ≤20 mm in diameter, lesions without ulcerative findings, lesions with negative horizontal margins, lesions with negative vertical margins, and lesions without lymphovascular invasion^6^. eCURA B is refer to the curability not requiring additional surgeries after endoscopic submucosal dissection **(**ESD).

eCURA C2 was defined as not applicable to eCURA B in undifferentiated-type cancers. eCURA C2) is refer to the curability requiring additional surgeries after ESD.

We compared the therapeutic outcomes between P-UDT lesions and M-UDT lesions treated with ESD (Table 3). Regarding factors that make a pre-treatment diagnosis difficult, M-UDT lesions were more likely than P-UDT to have a tumour diameter >20 mm, submucosal invasion, and the presence of ulcerative findings.

**Table 3:** Comparison of the therapeutic outcomes of endoscopic submucosal dissection for early gastric cancer between post-treatment pure undifferentiated-type lesions and post-treatment undifferentiated-type, predominant mixed-type lesions.

	Post-treatment P-UDT lesions (n = 258)	Post-treatment M-UDT lesions (n = 19)	P-value
Tumour diameter, mm	10 (6–15) 1–27	19 (12–25) 4–40	<0.0001
>20 mm	12 (4.7)	7 (36.8)	<0.0001
Invasion depth			0.0005
Intramucosal	240 (93.0)	12 (69.2)	
Submucosa	12 (4.76)	7 (36.8)	
Lymphovascular Invasion			
Presence of lymphatic invasion	3 (1.2)	1 (5.3)	0.2486
Presence of vascular invasion	0	1 (5.3)	0.0686
Positive vertical margin	4 (1.6)	0	>0.9999
Positive horizontal margin	7 (2.7)	0	>0.9999
Presence of ulcerative findings	8 (3.1)	6 (31.6)	<0.0001
En bloc resection	257 (99.6)	19 (100)	>0.9999

Data are presented as number (%), except tumour diameter, which is expressed as median (interquartile range) [range].

M-UDT, undifferentiated type predominant mixed type; P-UDT, pure undifferentiated type.

Next, we compared the pre-treatment factors (tumour diameter ≤20 mm or >20 mm, tumour depth, the presence or absence of ulcerations, tumour location, macroscopic appearance and histological type based on biopsy findings) between P-UDT and M-UDT lesions (Table 4). M-UDT for pre-treatment diagnosis based on biopsy findings was significantly more likely in M-UDT lesions than in P-UDT lesions.

**Table 4:** Comparison of post-treatment pure undifferentiated-type lesions and post-treatment undifferentiated-type, predominant mixed-type lesions considering the pre-treatment diagnosis.

Pre-treatment diagnosis	Post-treatment P-UDT lesions	Post-treatment M-UDT lesions	P-value
Tumour diameter			>0.9999
≤20 mm	258 (100)	19 (100)	
>20 mm	0	0	
Invasion depth			>0.9999
Intramucosal	258 (100)	19 (100)	
Submucosa	0	0	
Ulcerative findings			>0.9999
Absence	258 (100)	19 (100)	
Presence	0	0	
Location			0.1506
Upper third	25 (9.7)	4 (21.1)	
Middle third	168 (65.1)	13 (68.4)	
Lower third	65 (25.2)	2 (10.5)	
Macroscopic type			0.0567
Flat Depressed	254 (98.4)	17 (89.5)	
Others	4 (1.6)	2 (10.5)	
Pre-treatment histological type based on biopsy findings			<0.0001
P-UDT	258 (100)	12 (63.2)	
M-UDT	0	7 (36.8)	

Data are presented as number (%).

M-UDT, undifferentiated type predominant mixed type; P-UDT, pure undifferentiated type.

Finally, we compared the characteristics of lesions associated with the eCURA B and the eCURA C2 who underwent ESD for UDT EGC (Table 5). In cases of eCURA C2, a pre-treatment diagnosis of M-UDT was significantly more likely than a pre-treatment diagnosis of P-UDT.

**Table 5:** Comparison of lesion characteristics between the cases of eCURA B and the cases of eCURA C2 treated with endoscopic submucosal dissection for undifferentiated-type early gastric cancer.

	eCURA B (n = 229)	eCURA C2 (n = 48)	P-value
Location			0.2285
Upper third (%)	23 (10.0)	6 (12.5)	
Middle third (%)	146 (63.8)	35 (72.9)	
Lower third (%)	60 (26.2)	7 (14.6)	
Macroscopic type			0.0667
Flat Depressed (%)	226 (98.7)	45 (93.8)	
Others (%)	3 (1.3)	3 (6.3)	
Pre-treatment histology based on biopsy findings			0.0188
P-UDT (%)	226 (98.7)	44 (91.7)	
M-UDT (%)	3 (1.3)	4 (8.3)	

Data are presented as numbers (%).

M-UDT, undifferentiated type predominant mixed type; P-UDT, pure undifferentiated type.

eCURA B was defined in accordance with the Japanese guidelines for intramucosal undifferentiated lesions measuring ≤20 mm in diameter, lesions without ulcerative findings, lesions with negative horizontal margins, lesions with negative vertical margins, and lesions without lymphovascular invasion^6^. eCURA B is refer to the curability not requiring additional surgeries after endoscopic submucosal dissection (ESD).

eCURA C2 was defined as not applicable to eCURA B in undifferentiated-type cancers. eCURA C2) is refer to the curability requiring additional surgeries after ESD.

## Discussion

In this study, we clarified the differences in therapeutic outcomes of ESD for P-UDT cancers and M-UDT cancers and clarified whether a diagnosis of M-UDT correlated with requirement for additional surgeries. To our knowledge, this is the first study to investigate these relationships.

The cases of eCURA B were significantly higher for P-UDT lesions than for M-UDT lesions. Moreover, M-UDT lesions were more likely than P-UDT to have a tumour diameter >20 mm, submucosal invasion, and the presence of ulceration. In other words, because it is difficult to determine the tumour diameter, depth, and ulceration in the pre-treatment biopsy, when determining the indications for ESD in M-UDT, one should anticipate that eCURA B will occur more frequently in patients with P-UDT than in those with M-UDT lesions. Tumour diameter is determined based on the diagnostic demarcation of the tumour. We previously demonstrated the utility of magnifying narrow band imaging (ME-NBI) for diagnosis of demarcation in both ESD and surgical cases^16,17^. When the diagnostic accuracy of demarcation was assessed, the rate of diagnostic accuracy with ME-NBI was approximately 80%. In contrast, since the findings of UDT are mixed with the findings of DT, it is considered that findings of M-UDT are more complex than those of P-UDT. Therefore, in this study, it was considered that tumour diameter was more difficult to determine in M-UDT lesions than in P-UDT lesions. Based on the above, in M-UDT lesions, endoscopists should be more careful when performing diagnostic demarcation of the lesions to determine the accurate tumour diameter and not make a diagnosis based on the approximate tumour diameter indicated by negative biopsy results because of the difficulty in determining the tumour diameter for M-UDT lesions. Regarding the diagnosis of depth and ulcerative findings, the usefulness of endoscopic ultrasound (EUS) has been reported^18,19^. However, for UDT EGC, difficulty in diagnosis with EUS has been reported as well^18,19^. Therefore, further development of diagnostic modalities is warranted.

It is necessary to diagnose M-UDT lesions in biopsies correctly before treatment. Out of the M-UDT lesions diagnosed post-treatment, 36.8% had been diagnosed as M-UDT before treatment. Therefore, it is important to consider the diagnostic ability of other modalities.

It is possible to diagnose both DT^19,20^ and UDT EGC^16,21–23^ based on the characteristic findings of ME-NBI. We previously demonstrated the additive effect of combining ME-NBI and biopsy findings to determine pre-treatment histological type in MT-EGCs^24^. In this report, among all M-UDT lesions, both DT findings and UDT findings were recognised in 72.2% of the lesions. Since P-UDT has no DT findings, this result can be applied to distinguish between M-UDT and P-UDT. Although a biopsy can be used to make a focal diagnosis, we believe that ME-NBI may diagnose the entire lesion after determining the presence or absence of UDT or DT characteristics. Therefore, in the pre-treatment diagnosis of UDT lesions, it is suggested that a combination of ME-NBI and biopsy findings be used. In this study, in the cases of eCURA C2, a pre-treatment diagnosis of M-UDT was significantly more likely than a pre-treatment diagnosis of P-UDT. This combination may reduce misdiagnoses based on incorrect tumour diameters and lead to the avoidance of additional surgeries after ESD. In the future, prospective multicentre collaborative studies are necessary to investigate the differences in endoscopic findings of P-UDT and M-UDT lesions, and the development of another pre-treatment diagnostic modality is important.

This study has some limitations. First, it was a single-centre, retrospective study. Second, at present, it is unclear if any other modality can be used to improve the accuracy of the pre-treatment histological diagnosis. Third, there are only 19 cases with M-UDT, and thus the statistical power was limited. Finally, the results may change depending on the biopsy specimen. Although there were some limitations, our hospital is a high-volume centre specialised in cancer management, and cases treated over a 12-year period were used for this study. Therefore, the study data are considered sufficient to provide a historical basis for developing diagnostic modalities in the future.

In conclusion, the eCURA B outcome was higher for P-UDT lesions than for M-UDT lesions, and in patients with an eCURA C2 outcome a pre-treatment diagnosis of M-UDT was significantly more likely than a pre-treatment diagnosis of P-UDT. In patients with M-UDT lesions, pre-treatment diagnosis and careful consideration are necessary to determine the indications for ESD, thus, avoiding additional surgeries after ESD.

## Materials and Methods

### Patients

We retrospectively extracted objective information from electronic medical records, and analysed the records of all patients with pre-treatment biopsies diagnosed as UDT EGC after ESD (as initial treatment), at the Cancer Institute Hospital between May 2005 and March 2017. For pre-treatment diagnosis, we included lesions with tumour diameter ≤20 mm, intramucosal location, and absence of ulcerations, according to the Japanese guidelines^7^. We excluded patients with remnant gastric cancer or gastric tube cancer, patients who wanted ESD despite lack of indication for ESD based on their pre-treatment diagnosis, and patients diagnosed with DT predominant lesions by pre-treatment biopsy.

Generally, lesions with mixed histology type include various combinations. However, for this study, all such lesions were handled collectively as lesions with mixed histology. Tubular adenocarcinoma (well-differentiated and moderately differentiated) and papillary adenocarcinoma according to the WHO classification (tub1, tub2, and pap according to the Japanese classification of gastric carcinoma) are DT components^5,6^. Poorly cohesive carcinoma, including signet ring cell carcinoma and other subtypes according to the WHO classification (sig and por2 according to the Japanese classification of gastric carcinoma) are UDT components according to Japanese guidelines^6^. Therefore, differences among tub1, tub2, and pap in the DT components, and those between sig and por2 in the UDT components were not considered different.

**Figure 2:**
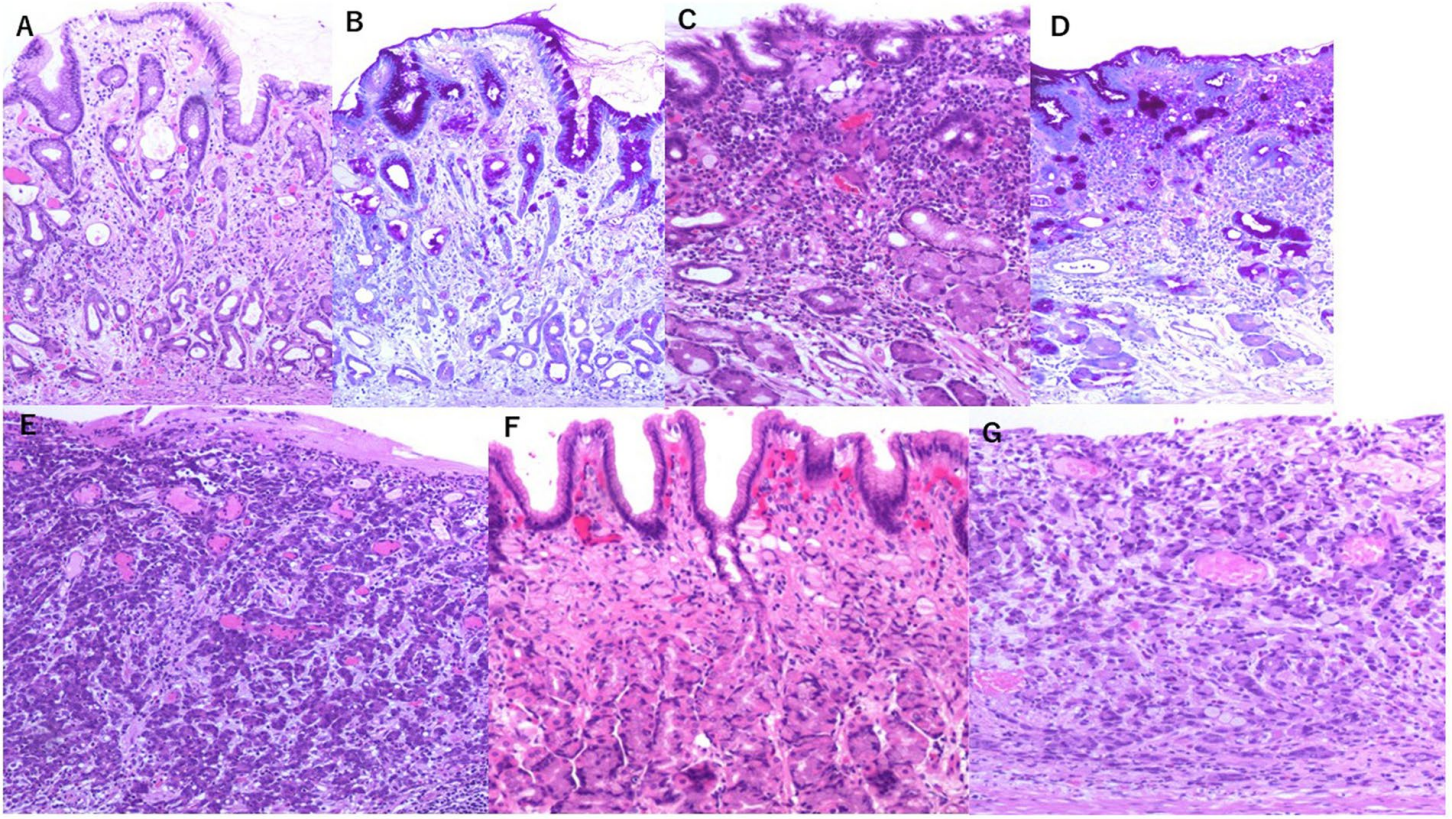
Histological images of undifferentiated-type gastric cancer. (**A**) Poorly differentiated adenocarcinoma (por2)-predominant mixed type [with tubular adenocarcinoma (tub2)] (Haematoxylin and eosin stain) (×100). (**B**) Poorly differentiated adenocarcinoma (por2)-predominant mixed type [with tubular adenocarcinoma (tub2)] (Periodic acid-Schiff stain) (×100). (**C**) Signet ring cell carcinoma (sig)-predominant mixed type [with tubular adenocarcinoma (tub2)] (Haematoxylin and eosin stain) (×100). (**D**) Signet ring cell carcinoma (sig)-predominant mixed type [with tubular adenocarcinoma (tub2)] (Periodic acid-Schiff stain) (×100). (**E**) Only poorly differentiated adenocarcinoma (por2) (pure undifferentiated type) (haematoxylin and eosin stain) (×100). (**F**) Only Signet ring cell carcinoma (sig) (pure undifferentiated type) (Haematoxylin and eosin stain) (×100). (**G**) Poorly differentiated adenocarcinoma (por2) and signet ring cell carcinoma (sig) (pure undifferentiated type) (haematoxylin and eosin stain) (×100). *According to the Japanese classification of gastric carcinoma^6^: Differentiated type; tubular adenocarcinoma, well-differentiated [tub1], tubular adenocarcinoma, moderately differentiated [tub2], and papillary adenocarcinoma [pap]. Undifferentiated type; signet ring cell carcinoma [sig], and poorly differentiated adenocarcinoma (non-solid type) [por2].

Based on the above, M-UDT was defined as a case in which the UDT component exceeded 50% of the lesion, and P-UDT was defined as a case comprising of an UDT component alone (Fig. 2).

### Study design and data collection

During this single-centre retrospective study, we first identified all UDT EGC patients treated during the study period and collected data on patient characteristics (age and sex), lesion characteristics (location, macroscopic type, and histological type based on biopsy findings), and therapeutic outcomes of ESD (median tumour diameter, invasion depth, post-treatment histological type, the presence of lymphovascular invasion, presence of ulceration, positive vertical margin, positive horizontal margin, en bloc resection, histological type post-ESD, and number of eCURA B).

Second, to clarify the differences in the cases of eCURA B for P-UDT cancers versus M-UDT cancers, we compared post-treatment histological type between the cases of eCURA B and the cases of eCURA C2 treated with ESD for UDT EGC.

Again, we compared the therapeutic outcomes of ESD for EGC between post-treatment P-UDT lesions and post-treatment M-UDT lesions. To detect any differences in therapeutic outcomes, the P-UDT and M-UDT groups were compared in terms of the cases of eCURA B and other factors known to affect this outcome, including tumour diameter, invasion depth, presence of lymphovascular invasion, positive vertical margin, positive horizontal margin, presence of ulcerative findings, and presence of en bloc resection.

Furthermore, to clarify whether M-UDT can be diagnosed before treatment, we compared the pre-treatment factors (tumour diameter ≤20 mm or >20 mm, tumour depth, the presence or absence of ulcerative findings, tumour location, and the macroscopic type and histological type based on biopsy findings) between P-UDT and M-UDT.

Finally, in order to clarify whether a pre-treatment diagnosis of M-UDT correlated with requiring additional surgeries, we compared characteristics of the lesions (location, macroscopic type, and pre-treatment histology based on biopsy findings) between the cases of eCURA B and cases of eCURA C2 treated with ESD for UDT EGC.

### Procedures

Before ESD, all UDT EGC patients underwent conventional endoscopy with dye-spraying and magnifying endoscopy with ME-NBI to diagnose the extent and depth of the tumour. The main histological type was determined based on the results of the pre-treatment biopsy. For all cancers detected in patients managed at our hospital, we determined the main histological diagnosis based on the biopsy results. According to the Japanese guidelines^6^, intramucosal cancers with a diameter of ≤20 mm and without ulcerative findings were preoperatively considered to meet the criteria of the expanded indications for ESD. At our institution, there is a well-established consensus regarding the expanded indications for ESD for UDT EGC.

For each lesion, negative biopsy results were collected from four sites surrounding the lesion (5–10 mm from the tumour margin). Afterwards each resected ESD specimen was submitted for histological analysis. The specimens had been cut into 2-mm slices for pathological evaluation, which was always performed by pathologists specialised in gastroenterology. The maximum tumour diameter, invasion depth, histological type, ulcerative findings, lymphovascular invasion, horizontal margin, and vertical margin were evaluated. eCURA B was defined in accordance with the Japanese guidelines for intramucosal undifferentiated lesions measuring ≤20 mm in diameter, lesions without ulcerative findings, lesions with negative horizontal margins, lesions with negative vertical margins, and lesions without lymphovascular invasion^6^. eCURA C2 was defined as not applicable to eCURA B in UDT cancers.

### Research involving human participants and/or animals

The study was approved by the Institutional Review Board (IRB) of Cancer Institute Hospital (IRB number 2017–1033). This study was conducted in compliance with the principles of the Declaration of Helsinki and its later amendments. Before recording the data, all personal identification information was removed.

### Informed consent

Before treatment for UDT EGC, all patients were provided with a detailed explanation of the advantages and disadvantages of ESD, and written informed consent to undergo the procedure was obtained from each patient. Additionally, written informed consent for the use of pathological specimens and imaging data for research purposes was obtained from each patient.

### Statistical analysis

Fisher’s exact probability test was employed for comparison between the two groups. For tumour diameter and age, the F-test was performed to determine whether distributions exhibited equal variance between the two groups. If the F-test showed significance, the median, interquartile range, and overall range were calculated and analysed using the Mann-Whitney U test by tumour diameter and age. If the F-test showed no significance, the mean ± standard deviation was calculated and analysed using the t-test by tumour diameter and age. Statistical significance was set at p-value of <0.05 for both univariate and multivariate analyses. JMP version 13.2 (SAS Institute Inc., Cary, NC, USA) was used for analysis.

## Data Availability

The data are not available for public access because of patient privacy concerns, but they are available from the corresponding author on reasonable request.
